# Comparative Proteomics of Milk Fat Globule Membrane Proteins from Transgenic Cloned Cattle

**DOI:** 10.1371/journal.pone.0105378

**Published:** 2014-08-18

**Authors:** Shunchao Sui, Jie Zhao, Jianwu Wang, Ran Zhang, Chengdong Guo, Tian Yu, Ning Li

**Affiliations:** 1 State Key Laboratory of Agrobiotechnology, China Agricultural University, Beijing, PR China; 2 Beijing GenProtein Biotech Company Ltd., Beijing, PR China; 3 Wuxi Kingenew Biotechnology Co., Ltd., Wuxi, Jiangsu Province, China; Università della Calabria, Italy

## Abstract

The use of transgenic livestock is providing new methods for obtaining pharmaceutically useful proteins. However, the protein expression profiles of the transgenic animals, including expression of milk fat globule membrane (MFGM) proteins, have not been well characterized. In this study, we compared the MFGM protein expression profile of the colostrum and mature milk from three lines of transgenic cloned (TC) cattle, i.e., expressing recombinant human α-lactalbumin (TC-LA), lactoferrin (TC-LF) or lysozyme (TC-LZ) in the mammary gland, with those from cloned non-transgenic (C) and conventionally bred normal animals (N). We identified 1, 225 proteins in milk MFGM, 166 of which were specifically expressed only in the TC-LA group, 265 only in the TC-LF group, and 184 only in the TC-LZ group. There were 43 proteins expressed only in the transgenic cloned animals, but the concentrations of these proteins were below the detection limit of silver staining. Functional analysis also showed that the 43 proteins had no obvious influence on the bovine mammary gland. Quantitative comparison revealed that MFGM proteins were up- or down-regulated more than twofold in the TC and C groups compared to N group: 126 in colostrum and 77 in mature milk of the TC-LA group; 157 in colostrum and 222 in mature milk of the TC-LF group; 49 in colostrum and 98 in mature milk of the TC-LZ group; 98 in colostrum and 132 in mature milk in the C group. These up- and down-regulated proteins in the transgenic animals were not associated with a particular biological function or pathway, which appears that expression of certain exogenous proteins has no general deleterious effects on the cattle mammary gland.

## Introduction

The technology of using genetically modified bovine lines to produce recombinant proteins in milk has flourished since the 1990s, based on the historic breakthrough of somatic cell cloning technology [1], [2]. Milk has been proven to be an excellent vehicle for producing and delivering recombinant human proteins [3]. The cow mammary gland can be modified to synthesize foreign proteins in transgenic cloned bovine milk, which may be useful for human consumption and assimilation because of its similarities of composition to human milk [4]. The advantages of milk-based system also include the easiness of animal housing and maintenance and lower cost of harvesting proteins than the cell bioreactor [5]. However, we have little information about how transgenic modification and cloning process influence bovine endogenous gene expression, milk characteristics and health. A further consideration is how these changes of products from these animals might affect the health of consumers. To address these issues, we examined both the protein expression profiles and composition of milk from transgenic cloned cows compared with conventionally bred animals. Many works have been done to analyze the compositions of meat and milk from transgenic cloned cattle, but the majority of these studies have focused on a few major constituents rather than a comprehensive analysis of proteins expression, especially of low-abundance proteins [6]–[8].

The concerns about consumption of food from transgenic cloned animals were the hot spot in recent research. The FDA has raised the instructions for the food from cloned animal and the studies have showed that the milk and meats from cloned cattle were as safe as the conventionally bred cattle. However, there were no instructions for food products from transgenic cloned animals, we have to use the instructions of cloned animals to examine the food products from transgenic cloned cattle. Milk proteins can be divided into three classes, namely casein, whey proteins, and MFGM proteins. Compared to other classes, MFGM proteins are the least abundant, making up 1–4% of total milk proteins, but they include thousands of different proteins [9], which were important composition of the milk proteins.

The MFGM is a protein-rich lipid bilayer that surrounds lipids in milk. Although the MFGM has been studied for 50–60 years, previous studies have focused primarily on membrane globule formation, intracellular transition, and secretion [10]. It is believed that the MFGM originates from the apical plasma membrane of mammary epithelial cells [11], [12]. Precursor microlipid droplets from the endoplasmic reticulum fuse to each other and travel to the apical cytoplasm where they are surrounded by the apical plasma membrane and then are secreted into the alveolar lumen [10], [13]–[15]. Therefore, we can obtain information about mammary epithelial cell health by analyzing the protein composition of the MFGM.

In addition, MFGM proteins have many beneficial bioactivities, e.g., as antibiotics and anticancer agents, MUC1 is one of the most abundant proteins in the MFGM and is thought to protect exposed surfaces from physical damage and invasive pathogenic microorganisms [16], [17]. For example, bovine lactophorin C-terminal fragment and PAS6/7, both MFGM constituents, inhibit replication of human rotavirus and prevent gastroenteritis [18]. MFGM proteins also have commercial value in cheese and butter production.

Technological advances in proteomics, especially quantitative proteomics, have enabled an increased understanding and characterization of milk proteins [19]. So far, proteomics has been successfully applied to studies of human milk whey proteins, bovine milk whey proteins, human MFGM proteins, bovine MFGM proteins, and goat MFGM proteins [20]–[23].

We genetically engineered three transgenic bovine lines that specifically express recombinant human α-lactalbumin (TC-LA group), lactoferrin (TC-LF group), and lysozyme (TC-LZ group) [24]–[26], from which the compositions of the milk include the human proteins with higher expression level compared to that from conventionally bred animals. Prior research for milk whey proteins and the nutritional components from transgenic cloned cattle have indicated that the expression of exogenous proteins did not significantly change the milk whey protein profile, and the mean values for the majority of the measured parameters were within the normal range [27].

In this work, we compared the MFGM proteins of colostrum and mature milk from these three healthy transgenic cloned lines to those from cloned and conventionally bred animals. The MFGM proteins of the five groups were characterized using 2D-PAGE and 2D LC-MS/MS coupled with the iTRAQ proteomic strategy. Our objectives were to: (1) characterize the proteins of the MFGM in transgenic cloned cattle; (2) compare the relative expression of MFGM proteins among transgenic cloned cattle milk; and (3) investigate the health of mammary epithelial cells in the transgenic cloned cattle. The study will supplement the former study by completing the evaluation for the milk products from transgenic cloned cattle and revealing the situation of the mammary epithelial cells from transgenic cloned cattle. This study provides the complement evidence of the milk composition analysis for the transgenic cloned bovine, and the results may indirectly reflect the health of the mammary epithelial cells of the transgenic cloned bovine.

## Materials and Methods

### Milk Sample Collection

The protocol was approved by the Institutional Animal Care and Use Committee of China Agricultural University (ID: SKLAB-2010-05-01). The three transgenic cattle lines TC-LZ (n = 10), TC-LA (n = 4), and TC-LF (n = 3) have been described [24]–[26] and were compared to cloned cattle (C, n = 3) which were also cloned by somatic cell nuclear transfer (SCNT), and conventionally bred cattle (N, n = 9) of a similar genetic background. The cattle were similar in age and lactation period and were housed under the same conditions. The milk collection were carried out as described previously [27]. The colostrum was obtained during the initial three days of lactation and mature milk was obtained on the 30^th^, 60^th^ and 90^th^ day after lactation and the milk was collected from the cows twice daily and was pooled to form one daily sample. Milk samples were collected by milking machine, and samples were immediately stored at −20°C until further analysis.

### Protein Extraction

Milk sample (50 ml) from each cow was centrifuged at 4000×g to separate milk fat globule cream from whole milk. The separated cream was rinsed in 25 ml phosphate buffered saline twice and 25 ml deionized water once. Then the milk fat globule proteins were obtained by methanol/trichloromethane precipitation as follows. The cream (2 ml) was then stored at –80°C, and upon thawing 10 ml methanol and 10 ml trichloromethane were added. The samples were centrifuged at 50,000×g for 30 min at 4°C (Avanti J-26XP, Beckman Coulter, Indianapolis, USA), and the supernatant was discarded. The precipitated proteins were solubilized in a solution containing 7 M urea, 2 M thiourea, 0.025 M DTT, 2% (w/v), and 1% CHAPS [20]. The relative concentration of protein in each extract was determined by measuring the amount of whey protein with the 2D-Quant quantitate kit (GE Healthcare). MFGM protein samples from each cow were pooled together to generate one individual sample and then individual samples in the same groups (TC-LZ, TC-LA, TC-LF, C and N) were pooled again to generate one group sample according to the equal protein mass.

### In-solution digestion

Soluble proteins (200 µg) were digested in-solution using the filter aided sample preparation (FASP) method [28]. Each protein extract (30 µl) was mixed with 200 µl of buffer containing 8 M urea in 0.1 M Tris-HCl (pH 8.5) in the filter unit and centrifuged at 14,000×g for 40 min and repeated one time. The flow-through was discarded, and iodoacetamide solution was added to the filter and mixed at 600 rpm for 1 min then incubated without mixing for 5 min. The filtered units were centrifuged at 14,000×g for 30 min, then 100 µl of buffer containing 8 M urea in 0.1 M Tris-HCl (pH 8.0) was added to the filtered unit, then the solution was centrifuged again at 14,000×g for 40 min and repeated twice. We then added 40 µl of the above-mentioned buffer containing endoproteinase Lys-C (enzyme to protein ratio 1∶50, w/w) and mixed at 600 rpm in thermo-mixer for 1 min. The units were incubated overnight then transferred to new collection tubes, and then 120 µl of 0.05 M NH_4_HCO_3_ in water with trypsin (enzyme to protein ratio 1∶100 w/w) was added and mixed at 600 rpm for 1 min. The units were incubated at room temperature for 4 h and then centrifuged at 14,000×g for 40 min. Finally, 50 µl of 0.5 M NaCl was added and the units were centrifuged at 14,000×g for 20 min.

The extracted peptides were desalted using a 1.3-ml C18 solid-phase extraction column (Sep-Pak® cartridge; Waters Corporation, Milford, USA). The peptides were dried using a vacuum centrifuge and then resuspended in 5 mM ammonium formate containing 5% acetonitrile, pH 3.0.

### LC-MS/MS Analysis

LC-MS/MS was performed on a nano Acquity UPLC system (Waters Corporation) connected to an LTQ Orbitrap XL mass spectrometer (Thermo Electron Corp., Bremen, Germany) equipped with an online nano-electrospray ion source (Michrom Bioresources, Auburn, USA). The MS was operated in data-dependent mode to switch automatically between MS and MS/MS acquisition. Survey full-scan MS spectra (m/z 300–1800) were acquired in the Obitrap with one microscan and with a mass resolution of 60,000 at m/z 400, followed by MS/MS of the eight most-intense peptide ions in the LTQ analyzer. LC-MS/MS spectra were acquired using SEQUEST (v.28 revision 12, Thermo Electron Corp) against the International Protein Index bovine database version 3.54. A decoy database containing the reverse sequences was appended to the database. The search parameters were set as follows: partial trypsin cleavage with two missed cleavage sites was considered; the peptide mass tolerance was 1.4 Da; and the fragment ion tolerance was 1 Da. Trans Proteomic Pipeline software (revision 4.20; Institute of Systems Biology, Seattle, WA) was then used to identify proteins based on corresponding peptide sequences with ≥95% confidence. The peptide results were filtered by PeptideProphet with a p value>0.90 and a ProteinProphet probability = 0.95 [29], [30].

### Quantitative Analysis of MFGM Proteins by iTRAQ LC-MS/MS

The digested peptides of the five groups were transferred to vials containing individual iTRAQ reagents (Applied Biosystems) following the iTRAQ standard protocol for the 8-plex kit. The N group was labeled with iTRAQ113, the TC-LF with iTRAQ114, TC-LZ with iTRAQ115, TC-LA with iTRAQ116, and the C group with iTRAQ117.

The iTRAQ-labeled samples were pooled and the SCX HPLC experiment was performed on a Shimadzu 20AD 5-µm SCX polysulfoethyl column (2.1 mm×100 mm, The Nest Group, Inc., MA) as the first dimension. Each collected components of the processed SCX fractions ran via RP-LC ESI-MS/MS on an Applied Biosystems Q-Star Elite XL mass spectrometer in which the RPLC column was a ZORBAX 300SB-C18 (5 µm, 0.1 mm×150 mm, Microm, USA). The Q-Star Elite XL mass spectrometer was operated in the smart information-dependent acquisition activated mode with automatic collision energy and automatic MS/MS accumulation. Survey full-scan MS spectra (m/z 400–1800) were acquired with one microscan and a mass resolution of 60,000 at m/z 400, followed by MS/MS of the four most-intense peptide ions in the analyzer. The relative abundance of the MFGM proteins in the different samples was derived from the ionic peak areas of the iTRAQ reporter.

iTRAQ identification and quantification analysis of the MFGM proteins were obtained using Protein Pilot 3.0 (Applied Biosystems, USA) with the following user-defined parameters: Sample Type, iTRAQ 8-plex (Peptide Labeled); Cysteine alkylation, MMTS; Digestion, Trypsin; Specify Processing, Quantitate; Database, IPI v3.62 bovine; Search Effort, thorough ID; Results Quality, Detected Protein Threshold [Unused ProtScore (Conf)] >1.3 (95%); Run False Discovery Rate Analysis.

## Results

### Overview of MFGM Proteome in the TC Groups

**Figure 1:**
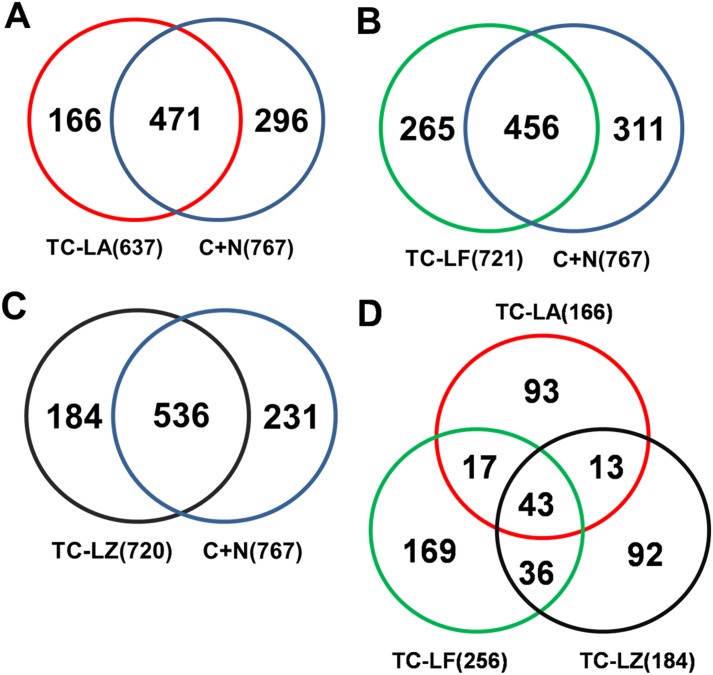
Comparison of MFGM proteins from the different TC and control lines. Cloned and conventionally bred control animals **[(C+N)]** were compared with (A) MFGM proteins from the TC-LA line, (B) MFGM proteins from the TC-LF line, and (C) MFGM proteins from the TC-LZ line. (D) The intersection of differentially expressed MFGM proteins from the three TC groups yielded 43 proteins in common.

**Figure 2:**
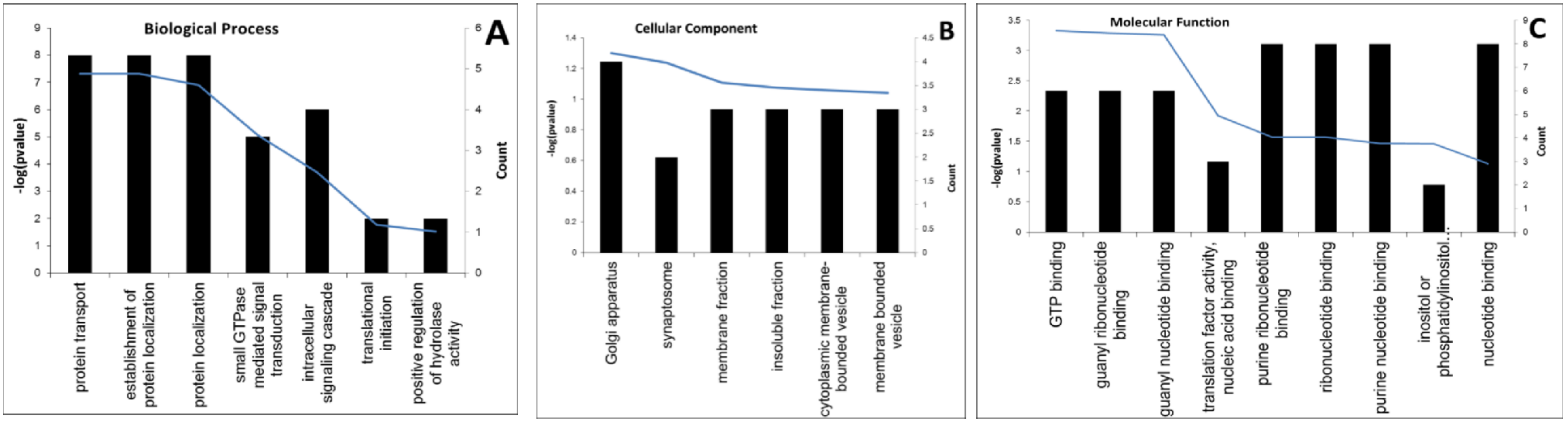
Functional analysis of MFGM proteins specifically expressed in the transgenic cloned animals. Analysis of proteins enriched in the transgenic cloned groups according to: (A) biological process, (B) cellular component, and (C) molecular function. Blue curves indicate the degree of enrichment of gene function.

MFGM proteins were characterized in three TC bovine lines and in C group and N group using 2D nano-LC-MS/MS and a quantitative proteomics method (iTRAQ). LC-MS/MS identified 1225 proteins among the five groups of MFGM samples, 939 proteins in colostrum, and 910 proteins in mature milk (Table 1). There were 637 proteins that were present in TC-LA group, 721 in the TC-LF group, 720 in the TC-LZ group, 527 in the C group, and 668 in the N group. iTRAQ identified 851 MFGM proteins in colostrum and 775 MFGM proteins in mature milk of all the bovine lines. The identified proteins from the three TC groups were compared to those from the C and N groups. The TC-LA group contained 166 proteins that were not found in the control lines, the TC-LF group contained 265 proteins, and the TC-LZ group contained 184 proteins (Figure 1). We found that 43 of these proteins were shared by all the three TC groups (Table 2). The molecular mass of this subset was between 10 kDa and 90 kDa, and the pI values were between 4 and 10. DAVID Bioinformatics Resources was used to analyze the function of the 43 common proteins in GO terms [31]. We found that these proteins were enriched in the biological processes of protein transport, cellular component of the Golgi apparatus, and the molecular function of GTP binding. However, the functions of these specific expressed proteins did not cluster in any particular pathway (Figure 2).

**Table 1:** Proteins identified in the MFGM of colostrum and mature milk of the TC-LZ, TC-LA, and TC-LF transgenic cloned lines and of the cloned and conventionally bred normal controls.

	TC-LZ	TC-LA	TC-LF	Cloned	Normal	Total
**Colostrum**	620	516	411	220	562	939
**Mature milk**	444	393	567	464	353	910
**In total**	720	637	721	527	668	1225

**Table 2:** Proteins specifically expressed in transgenic cloned cattle MFGM.

No	Accession	Description	MW	PeptideLength	IsoelectricPoint
1	IPI00686092.1	Peroxiredoxin-1	22194.98	199	8.6084
2	IPI00687416.2	Rras2 Protein	23384.41	204	5.8076
3	IPI00687550.1	3′(2′),5′-BisphosphateNucleotidase 1	33306.63	308	5.1936
4	IPI00689149.2	Oxysterol-Binding Protein	84236.69	746	6.2374
5	IPI00692733.2	Transmembrane Protein 30A	40656.91	361	8.6073
6	IPI00693697.3	Similar To Adiponutrin	50423.45	455	7.5812
7	IPI00695308.5	Mucin-16 (Fragment)	31335.98	282	9.0396
8	IPI00695670.1	Upf0585 Protein C16Orf13 Homolog	22699.17	204	7.9092
9	IPI00695741.2	62 Kda Protein	62274.23	569	5.3891
10	IPI00696647.1	Similar To Pincher Isoform 1	60985.31	540	7.6711
11	IPI00697565.3	Ras-Related Protein Rab-21	24130.66	222	8.0481
12	IPI00698430.1	Eukaryotic Translation InitiationFactor 5 Isoform 7	48939.49	429	5.2609
13	IPI00700391.1	Similar To V-Crk SarcomaVirus Ct10 OncogeneHomolog Isoform 1	33809.31	304	5.2451
14	IPI00703351.2	Similar To Aldehyde Dehydrogenase3B1 Isoform 1	53661.38	486	8.4692
15	IPI00703423.1	B-Cell Receptor-Associated Protein 31	27883.64	245	9.765
16	IPI00704666.1	Trna Methyltransferase 112 Homolog	14309.9	125	5.8553
17	IPI00704752.1	Ras-Related Protein Rab-7A	23528.72	207	6.5881
18	IPI00706451.3	Adp-Ribosylation Factor 4	20514.36	180	5.9819
19	IPI00706632.1	Elongation Factor 1-Gamma	50345.45	440	6.5236
20	IPI00706968.3	Nuclear Factor Of KappaLight Polypeptide Gene EnhancerIn B-Cells Inhibitor, Beta	37543.32	357	4.2979
21	IPI00707103.4	Synaptosomal-Associated Protein 29	28471.85	258	5.1001
22	IPI00707616.3	Cidea Protein	24524.03	219	9.7273
23	IPI00708591.1	Znrf2 Protein	23870.75	238	7.1119
24	IPI00710727.1	Transitional Endoplasmic Reticulum Atpase	89272.24	806	4.8908
25	IPI00711304.4	Similar To InositolPolyphosphate-5-Phosphatase A	70475.79	637	8.6043
26	IPI00713743.1	Inosine-5′-MonophosphateDehydrogenase 2	55726.54	514	7.339
27	IPI00714515.1	Fgr Protein	59292.95	527	5.3694
28	IPI00714621.3	Uncharacterized ProteinC1Orf93 Homolog	21482.83	201	6.2609
29	IPI00714818.1	Eukaryotic Translation InitiationFactor 3 Subunit H	39879.94	352	6.653
30	IPI00714992.4	Cgmp-Dependant Type Ii Protein Kinase	87033.26	762	8.6701
31	IPI00715153.2	Fas-Associated Factor 2	52629.57	445	5.4387
32	IPI00716638.7	Wd Repeat-ContainingProtein 1	66214.06	606	6.7147
33	IPI00717376.3	Similar To Cytoskeleton-AssociatedProtein 4	64489.56	589	5.9276
34	IPI00718291.4	Rab14 Protein	23881.49	215	6.1266
35	IPI00718671.5	Synaptosomal-Associated Protein	23212.11	211	4.4574
36	IPI00729755.2	Acyl-Coa Synthetase Long-ChainFamily Member 1	78228.82	699	7.2276
37	IPI00730045.3	Similar To Rififylin Isoform 4	39655.22	356	6.1033
38	IPI00733615.1	Dnaj Homolog SubfamilyA Member 2	45731.44	412	6.4421
39	IPI00867128.1	Phosphoserine Aminotransferase	40502.07	370	7.7533
40	IPI00867311.1	Scamp2 Protein	36678.97	328	6.1107
41	IPI00906554.1	Guanine Nucleotide-BindingProtein Subunit Alpha-11	42042.75	359	6.029
42	IPI00924133.1	Similar To Ciliary NeurotrophicFactor Receptor Alpha Precursor(Cntfr Alpha) Isoform 3	40661.3	372	6.9436
43	IPI00944416.1	Cyb5B Protein	16267.5	146	4.8233

### Quantitative Comparison of MFGM Proteins

**Figure 3:**
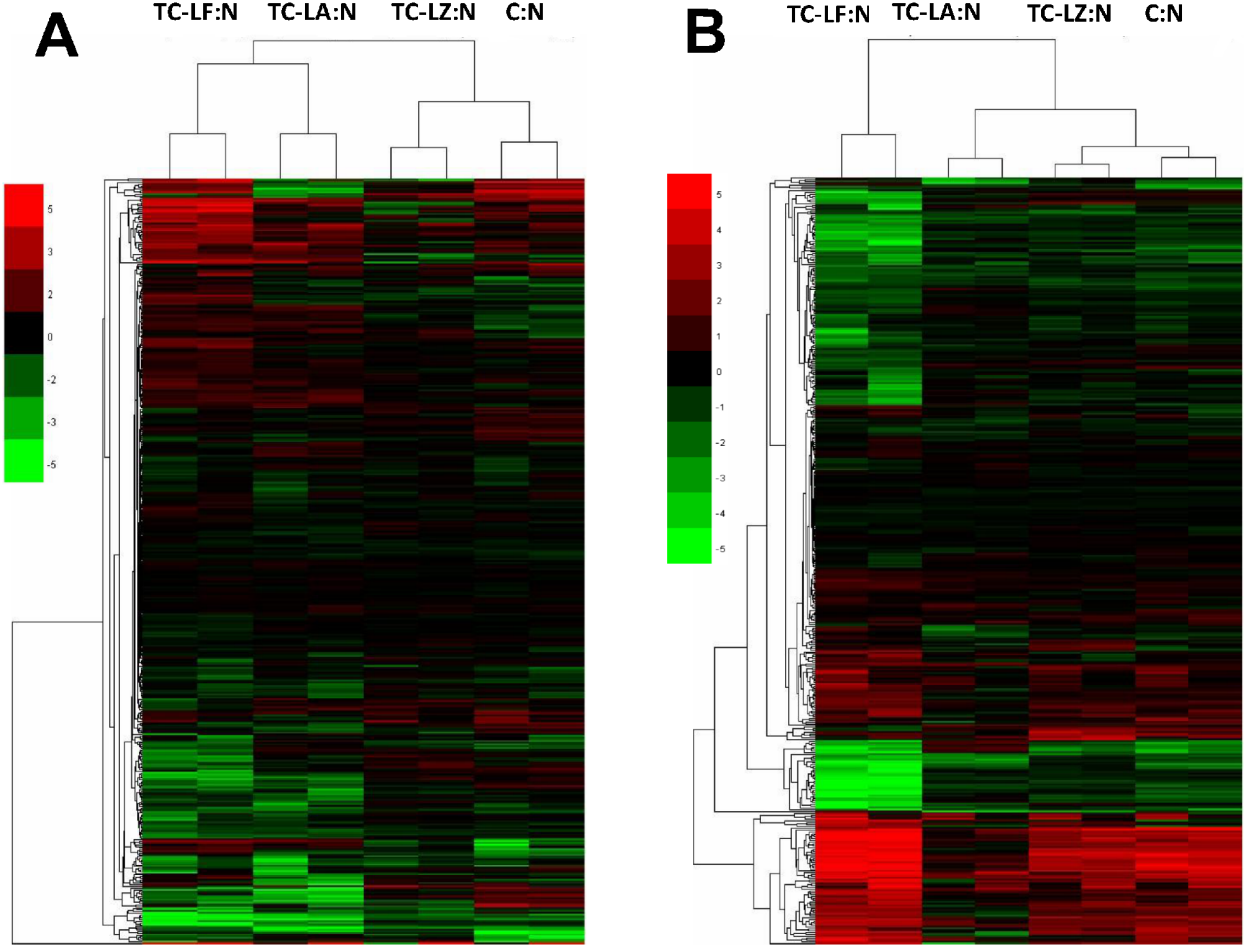
Cluster analysis of MFGM proteins. (A) Analysis of MFGM proteins in colostrum of the three TC groups compared to cloned **[(C)]** and conventionally bred **[(N)]** controls. (B) Analysis of MFGM proteins in mature milk of the three transgenic groups compared to controls.

We obtained relative MFGM protein quantity information from the five groups using iTRAQ. Only proteins with consistent quantitative results in two independent iTRAQ runs were used for further analysis of expression and function. A total of 459 proteins from colostrum MFGM and 426 proteins from mature MFGM were identified. Statistical analysis of expression levels in the three TC groups and C group compared with the N group revealed more than twofold differences (p≤0.05) in the expression of many of these proteins (Figure 3). In this regard, 126 proteins in the colostrum and 77 proteins in the mature milk of the TC-LA group had a greater than twofold difference in expression, likewise, in the TC-LF group there were 157 proteins in colostrum and 222 proteins in mature milk, in the TC-LZ group there were 49 proteins in colostrum and 98 proteins in mature milk, and in the cloned control group there were 98 proteins in colostrum and 132 proteins in mature milk.

**Figure 4:**
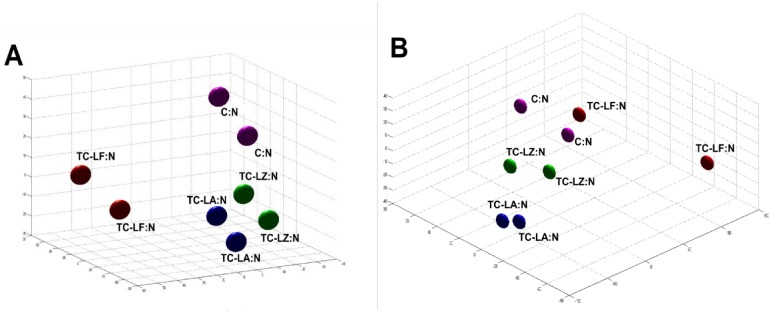
Principal component analysis of MFGM proteins from the three transgenic groups, the cloned group, and the conventionally bred control group. (A) Analysis of MFGM proteins in colostrum. (B) Analysis of MFGM proteins in mature milk.

Comparison between the MFGM proteins among the five groups also revealed many differences. The unsupervised hierarchical cluster analysis (HCA) and the principal component analysis (PCA) were used to assess the variability among the 5 groups. We found that the TC-LA, TC-LZ, and C groups had similar protein compositions in colostrum and mature milk. The TC-LF group was distinguishable from the other two TC groups (Figure 4). The colostrum of the TC-LF and TC-LA groups clustered together as that of the TC-LZ and C groups did. The results indicated that in the colostrum the TC-LZ was closer to the C group and in the mature milk the TC-LZ and TC-LA were closer to the C group. Both in colostrum and mature milk, the TC-LF groups were showed a greater difference from the other TC groups. In colostrum, 11 proteins were differentially expressed in the TC-LA group compared to the control groups, 25 in the TC-LF group, and 6 in the TC-LZ group. In mature milk, there were 8 proteins that were differentially expressed compared to the controls in the TC-LA group, 25 in the TC-LF group, and 5 in the TC-LZ group. In TC groups, the proteins with expression changed simultaneously were not appeared. Several of these differentially expressed proteins, such as fibrinogen α chain, α-S2-casein, κ-casein, β-lactoglobulin, and lactoferrin have been explored in other studies of whey and MFGM [20], [32]–[34].

Our identified MFGM proteins were subjected to cluster analysis to evaluate the relationship between TC animals and conventionally bred controls. In the TC-LA group, six proteins were notably down-regulated, including α-2-antiplasmin (−4.9 fold), β-lactoglobulin (−4.8 fold), serum albumin (−4.9 fold), platelet glycoprotein 4 (−4.1 fold), lactoferrin (−4.2 fold), serine hydroxymethyltransferase, mitochondrial (−4.1 fold), and a putative uncharacterized protein (−4.4 fold). In the TC-LF group, 10 proteins were notably different from the control group, including apolipoprotein A-II (−4.4 fold), ATP-binding cassette sub-family G (WHITE) member 2 (+4.3 fold), α-1-acid glycoprotein (−4.9 fold) similar to solute carrier family 39 (zinc transporter) member 8 (+4.1 fold), α-S2-casein (+4.2 fold), MUC 1 (+4.9 fold), acyl-CoA synthetase long-chain family member 1 (+4.8 fold), and vitamin D–binding protein (−5.2 fold). In the TC-LZ, four proteins were notably different from the control group, namely fibrinogen α chain (+2.8 fold), β-lactoglobulin (−2.7 fold), cidea protein (−3.1 fold), and similar to ATPase type 13A4 (−2.9 fold). In the C group, six proteins were notably different from the conventionally bred group, namely fibrinogen α chain (+3.1 fold), α-S2-casein (+5.3 fold), α-S1-casein (+4 fold), Rit1 protein (−3.6 fold), α-lactalbumin (−4.8 fold), and nucleobindin-1 (+3.3 fold).

### Function and Pathway Analysis of Differentially Expressed Proteins

**Figure 5:**
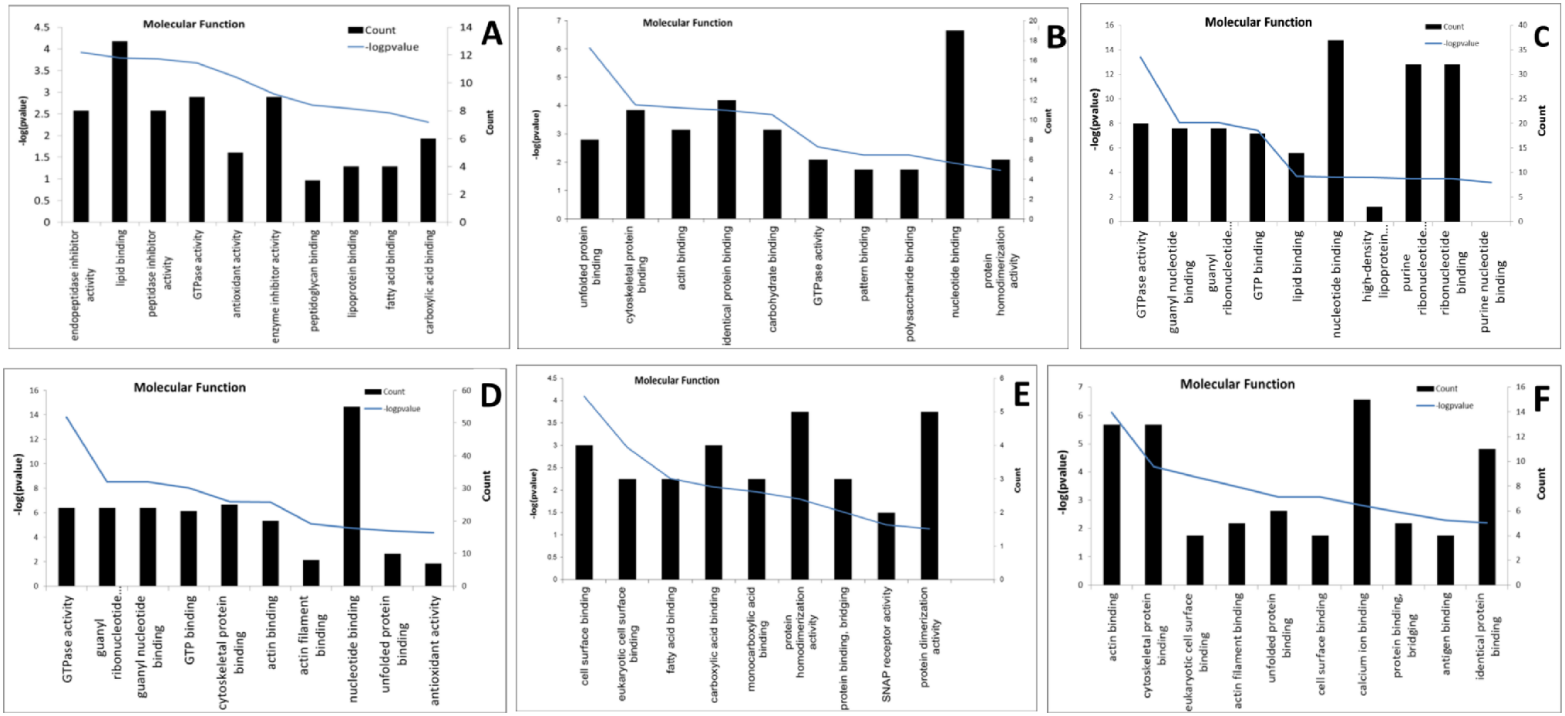
Molecular function analysis of differentially expressed MFGM proteins in the transgenic groups. Enrichment analysis of differentially expressed MFGM proteins in (A) TC-LA colostrum, (B) TC-LA mature milk, (C) TC-LF colostrum, (D) TC-LF mature milk, (E) TC-LZ colostrum, and (F) TC-LZ mature milk. Blue curves indicate the degree of enrichment of gene function.

The Ingenuity Pathway Analysis (IPA, Ingenuity Systems, Inc. Redwood City) software was used to analyze the functions and networks of the differentially expressed proteins. To further examine the specifically expressed proteins, they were categorized according to their biological process, cellular component or molecular function as annotated in the Gene Ontology (GO) database. Differentially expressed MFGM proteins from TC-LA colostrum were mainly enriched in the biological processes of response to wounding and the acute inflammatory response, in the cellular components of the extracellular region, and in the molecular function of endopeptidase inhibitor activity. Differentially expressed MFGM proteins from TC-LA mature milk were mainly enriched in cytoskeleton organization, membrane-bounded vesicles, and unfolded protein binding. Differentially expressed MFGM proteins from TC-LF colostrum were mainly enriched in response to organic substance, the extracellular region, and endopeptidase inhibitor activity. Differentially expressed MFGM proteins from TC-LF mature milk were mainly enriched in small GTPase–mediated signal transduction, pigment granules, and GTPase activity. Differentially expressed MFGM proteins from TC-LZ colostrum were mainly enriched in response to wounding, the extracellular region, and cell-surface binding. Finally, differentially expressed MFGM proteins from TC-LZ mature milk were mainly enriched in cell motility, the actin cytoskeleton, and actin binding (Figure 5).

**Figure 6:**
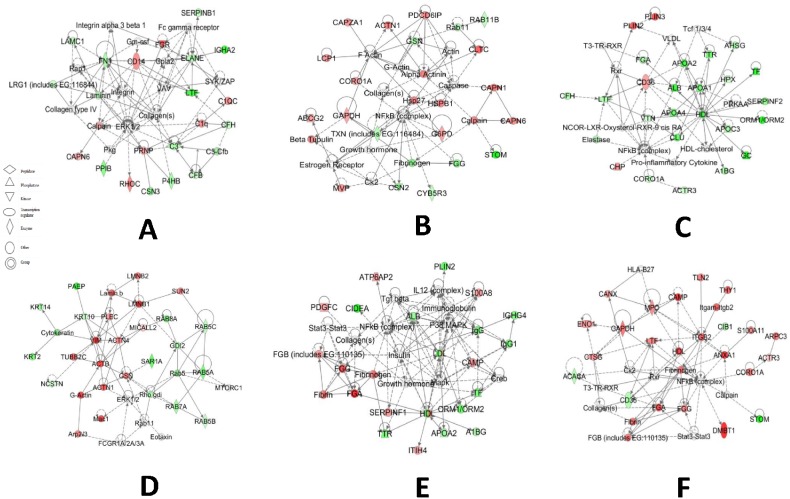
Pathway analysis of differentially expressed proteins from the transgenic groups. IPA defined pathways of the differentially expressed MFGM proteins from: (A) TC-LA colostrum, (B) TC-LA mature milk, (C) TC-LF colostrum, (D) TC-LF mature milk, (E) TC-LZ colostrum, and (F) TC-LZ mature milk.

In the TC-LA group, there were 18 proteins that were changed above 3 folds in colostrum and mature milk. The pathway analysis showed that the changed MFGM proteins in the TC-LA colostrum most involved in the network which have the functions of cellular movement, immune cell trafficking, cell-to-cell signaling and interaction. And in the mature milk are cell death, cell-to-cell signaling and interaction, hematological system development and function (Figure 6A, B).

In TC-LF group, there were 46 proteins that were changed in colostrum and mature milk significantly. The IPA software showed that the changed MFGM proteins in mature milk involved in the network of which top functions are cellular assembly and organization, cellular function and maintenance, dermatological diseases and conditions. And the MFGM proteins from colostrum involved in the network of which top functions are lipid metabolism, molecular transport, and small molecule biochemistry (Figure 6C, D).

In group of TC-LZ, there were 10 proteins that were altered obviously in colostrum and mature milk. The IPA software showed that the changed MFGM proteins in TC-LZ mature milk involved in the network of which the top functions are cell-to-cell signaling and interaction, tissue development, cell death. And in colostrum MFGM proteins, the top functions are inflammatory response, cell-to-cell signaling and interaction, cell signaling (Figure 6E, F).

**Figure 7:**
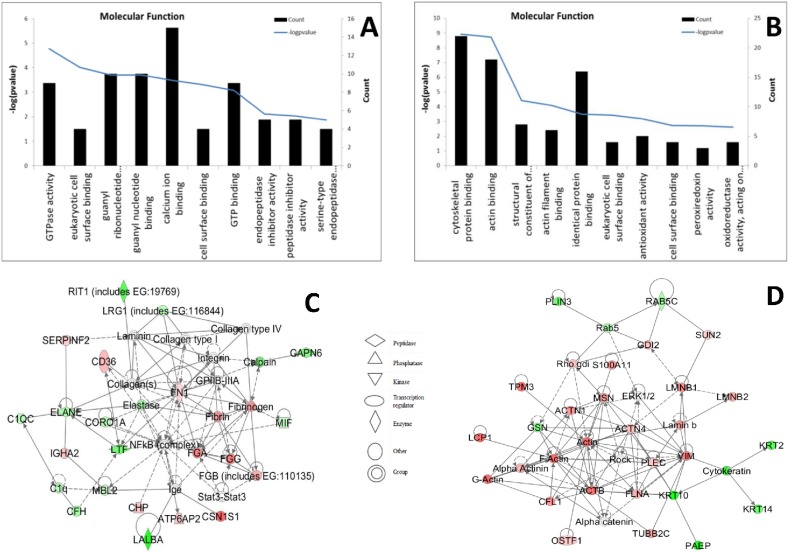
Molecular function and pathway analysis of differentially expressed MFGM proteins from the cloned control group. (A, B): Molecular function of MFGM proteins from colostrum (A) and mature milk (B). (C, D): IPA defined pathways of MFGM proteins from colostrum (C) and mature milk (D).

The MFGM proteins of C group were also compared with the N group, the results showed that the changed proteins in the colostrum involved in the functions of cell-to-cell signaling and interaction, tissue development, hematological system development and function. In mature MFGM, the changed proteins were in the network of functions: cellular movement, cellular assembly and organization, cellular function and maintenance. Differentially expressed MFGM proteins in colostrum from the C group were enriched in the biological processes of response to wounding and the acute inflammatory response, in the cellular components of the extracellular region, and in the molecular function of GTPase activity. Differentially expressed MFGM proteins in mature milk of the cloned control animals were enriched in the biological process of glucose catabolism, in the cellular component of the actin cytoskeleton, and in the molecular function of cytoskeletal protein binding (Figure 7).

**Figure 8:**
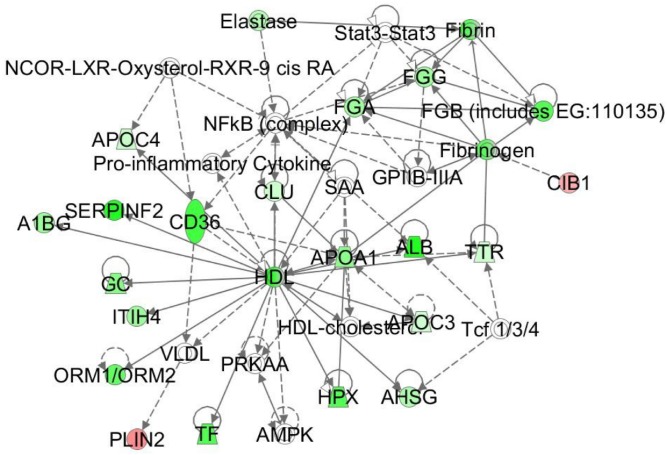
Pathway analysis involved in the Lipid Metabolisms of the TC-LA MFGM proteins.

## Discussion

Proteomics is one of the most popular methods to analyze complex samples. Recent advances in proteomics make the identification of low-abundance proteins and analysis of complex proteins such as high-molecular-weight transmembrane proteins possible, such analyses are not possible with standard 2D-PAGE methods alone. The combination of LC-MS/MS and iTRAQ or methods with the iTRAQ technologies are suitable for analysis of hydrophobic macro-molecules like plasma membrane proteins and are highly sensitive, fast, and reliable. Comparing with traditional 2D-PAGE method, the methods based on mass spectrometry are more suitable for MFGM proteins analysis. Data from several previous studies of MFGM proteins in humans [23], cattle [35] and goats [36] demonstrated that the amount of the identified proteins by this method was larger than other methods and the iTRAQ method was suitable to analyze the MFGM proteins with the same sensitivity compared to LC-MS/MS.

Applying these methods, we examined the effects of exogenous gene expression and cloning techniques on bovine MFGM proteins, an neglected area of research. Comparing three TC bovine lines expressing different exogenous proteins with C and N group, we identified 43 proteins that were specifically expressed in the transgenic animals below the detection limit for 2D-PAGE and silver staining. Considering both the concentration and functional analysis, we believe that these specific expressed proteins did not adversely influence the composition of milk or the health of these transgenic animals. Peroxiredoxin-1 was hypothesized to responsible for the anti-oxidative effect of laminar share stress [37]. 3′ (2′), 5′-bisphosphate nucleotidase 1, inosine-5′-monophosphate dehydrogenase 2, elongation factor 1-gamma, sdp-ribosylation factor 4 and phosphoserine aminotransferase involved in the metabolism of the cell, the protein ras-related protein rab-21 and guanine nucleotide-binding protein subunit alpha-11 involved in the mediated signal transduction. The function of b-cell receptor-associated protein 31 involves in apoptotic cleavage of cellular proteins, the function of ras-related protein rab-7A involves in protein transport, the function of acyl-coa synthetase long-chain family member 1 involves in the fatty acid degradation and metabolism, the function of Eukaryotic Translation Initiation Factor 3 Subunit H was in regulation of translational initiation.

There were distinguishable differences between the three transgenic lines in terms of exogenous protein expression. The TC-LF group expressed recombinant human lactoferrin at high levels, i.e., 2.5–3.8 g/l [25], the TC-LA group, expressed recombinant human α-lactalbumin at intermediate levels, i.e., 1.5 g/l [26], and the TC-LZ group expressed recombinant human lysozyme at low levels, i.e., 13–28 mg/l [24]. The expression profile of TC-LF group displayed a marked difference with that of other TC groups but the range of variation was comparable to the C group. Comprehensive analysis of the relative quantities of MFGM proteins in the transgenic and control groups led to the hypothesis that MFGM protein expression was influenced by the quantity of the exogenous proteins. In the TC-LF group, lactoferrin expression was higher than that of the exogenous proteins in the other two transgenic groups, and the number of the affected MFGM proteins was also higher. One possible mechanism for this could entail the S100 proteins, which are involved in inflammatory and antimicrobial responses. We found low abundance of the S100A8 and S100A9 proteins in colostrum of the transgenic animals relative to the N group. This may be a consequence of the fact that LF, LA, and LZ (i.e., which were expressed exogenously in our study animals) are all involved in the inflammatory response.

The MFGM proteins are important in cattle cultivation and quality of livestock products, especially in cheese industry. The MFGM proteins were the major composition in MFGM, they play a core role in mammary epithelial cell against bacteria, so these proteins and immune globulin proteins are highly expressed in colostrum MFGM, and the colostrum was beneficial to the acquired immunity in new born.

In TC-LA group, we analyzed the functions of the proteins and found out that plenty of them were not defined, such as similar to lipocalin, α-2-antiplasmin, Cd5L protein, putative uncharacterized protein, canx protein. Hemopexinand serotransferrin coming from blood majorly influenced by the cattle individually. Actin-related protein 2/3 complex subunit 3, rab35 protein, serine hydroxymethyltransferase, mitochondrial, l-lactate dehydrogenase B Chain are involved in ubiquitin ligase [38]. Apolipoprotein E plays a key role in regulating plasma levels of lipoproteins and its level was not associated with high-density lipoprotein fractions [39]. The former studies of the composition of milk from transgenic cloned cattle indicated that the fatty acids of TC-LA colostrum were less than that of the N group especially in monounsaturated fatty acids [27]. The pathway analysis found the down-regulated lipid metabolism (Figue 8), which presumably was involved in monounsaturated fatty acids synthesis.

In TC-LF group, some proteins have high abundance in milk such as κ-Casein, α-S2-Casein, serotransferrin, α-lactalbumin,serum albumin and lactoperoxidase, so a small amount of residual proteins will cause detectable change of concentrations. Plenty proteins with the function of lipid metabolism were differentially expressed. Bovine MUC1 prevents binding of bacteria to human intestinal cells and has a role in preventing the binding of common enteropathogenic bacteria to human intestinal epithelial surfaces [40]. The expression of fatty acids of mature TC-LF milk was less than that of other four groups, especially in saturated fatty acids [27]. Plenty proteins which involve in lipid metabolism were expressed lowly in TC-LF mature MFGM, such as apolipoprotein A-II, which is one of the major proteins in HDL and its main function is to modulate the lipid binding and lecithin-cholesterol acyltransferase activities of HDL by promoting the dissociation of apo A-I from HDL [41]. ATP-binding cassette, sub-family G (White), member 2 have the function of maintaining lipid homeostasis in the mammary gland [42]. Fatty acid-binding protein (FABP) regulates the channeling of fatty acids toward copious milk fat synthesis in bovine mammary gland [43]. The expression level of CIDE-A protein was regulated by insulin and/or fatty acids in mammary epithelial cells, and thereby played an important role in lipid and energy metabolism [44]. Acyl-coa synthetase long-chain family member 1 (ACSL1) which involves in fatty acid analysis [43] was differentially expressed in lactation,. ACSL1, FABP and CD 36 were suggested to have the function of channel long chain fatty acids toward esterification into milk triacylglycerol [43], [45].

The studies showed that both the bovine and human α-lactalbumin had the functions of feeds back on the mammary gland to regulate involution [46]. In our study, the bovine α-Lactalbumin and the exogenous human α-Lactalbumin were also expressed in mammary gland and the concentration was higher than the normal bovine. But we did not notice that the α-lactalbumin could induce the apoptotic activity, and our former study have confirmed the biological activity of recombination human α-lactalbumin [26]. In this study, over expression of the α-lactalbumin did not influence the expression of MFGM proteins, which indicated that the mammary gland epithelial cell would not be affected from the apoptotic activity.

In summary, the study uses proteomics methods to analyze transgenic cloned cattle MFGM proteins for the first time. The expression of exogenous proteins did not significantly change the MFGM protein profiles, and the relative quantity expression of MFGM proteins were all within the normal ranges. The differences among the TC groups were not greater than those between the N group and C group. The data from this study improves the understanding of the bovine milk proteome and provides data for the assessment of the food safety of transgenic cloned animals.

## References

[1] Simons JP, McClenaghan M, Clark AJ (1987) Alteration of the quality of milk by expression of sheep beta-lactoglobulin in transgenic mice. Nature 328: 530–532. 3614356 10.1038/328530a0

[2] Gordon K, Lee E, Vitale JA, Smith AE, Westphal H, et al (1992) Production of human tissue plasminogen activator in transgenic mouse milk. 1987. Biotechnology 24: 425–428. 1422049

[3] Rudolph NS (1999) Biopharmaceutical production in transgenic livestock. Trends Biotechnol 17: 367–374. 10461183 10.1016/s0167-7799(99)01341-4

[4] Houdebine LM (2000) Transgenic animal bioreactors. Transgenic Res 9: 305–320. 11131009 10.1023/A:1008934912555 PMC7089244

[5] Wheeler MB, Walters EM, Clark SG (2003) Transgenic animals in biomedicine and agriculture: outlook for the future. Anim Reprod Sci 79: 265–289. 14643108 10.1016/s0378-4320(03)00168-4

[6] Laible G, Brophy B, Knighton D, Wells DN (2007) Compositional analysis of dairy products derived from clones and cloned transgenic cattle. Theriogenology 67: 166–177. 17052749 10.1016/j.theriogenology.2006.09.028

[7] Baldassarre H, Hockley DK, Olaniyan B, Brochu E, Zhao X, et al (2008) Milk composition studies in transgenic goats expressing recombinant human butyrylcholinesterase in the mammary gland. Transgenic Res 17: 863–872. 18483775 10.1007/s11248-008-9184-5

[8] Baldassarre H, Schirm M, Deslauriers J, Turcotte C, Bordignon V (2009) Protein profile and alpha-lactalbumin concentration in the milk of standard and transgenic goats expressing recombinant human butyrylcholinesterase. Transgenic Res 18: 621–632. 19296233 10.1007/s11248-009-9254-3

[9] Quaranta S, Giuffrida MG, Cavaletto M, Giunta C, Godovac-Zimmermann J, et al (2001) Human proteome enhancement: high-recovery method and improved two-dimensional map of colostral fat globule membrane proteins. Electrophoresis 22: 1810–1818. 11425236 10.1002/1522-2683(200105)22:9<1810::AID-ELPS1810>3.0.CO;2-M

[10] Keenan TW (2001) Milk lipid globules and their surrounding membrane: a brief history and perspectives for future research. J Mammary Gland Biol Neoplasia 6: 365–371. 11547904 10.1023/a:1011383826719

[11] Keenan TW (2001) Assembly and secretion of the lipid globules of milk. Adv Exp Med Biol 501: 125–136. 11787674 10.1007/978-1-4615-1371-1_16

[12] Auty MA, Twomey M, Guinee TP, Mulvihill DM (2001) Development and application of confocal scanning laser microscopy methods for studying the distribution of fat and protein in selected dairy products. J Dairy Res 68: 417–427. 11694044 10.1017/s0022029901004873

[13] Mather IH, Keenan TW (1998) Origin and secretion of milk lipids. J Mammary Gland Biol Neoplasia 3: 259–273. 10819513 10.1023/a:1018711410270

[14] Heid HW, Keenan TW (2005) Intracellular origin and secretion of milk fat globules. Eur J Cell Biol 84: 245–258. 15819405 10.1016/j.ejcb.2004.12.002

[15] Wu CC, Howell KE, Neville MC, Yates JR 3rd, McManaman JL (2000) Proteomics reveal a link between the endoplasmic reticulum and lipid secretory mechanisms in mammary epithelial cells. Electrophoresis 21: 3470–3482. 11079566 10.1002/1522-2683(20001001)21:16<3470::AID-ELPS3470>3.0.CO;2-G

[16] Patton S, Gendler SJ, Spicer AP (1995) The epithelial mucin, MUC1, of milk, mammary gland and other tissues. Biochim Biophys Acta 1241: 407–423. 8547303 10.1016/0304-4157(95)00014-3

[17] Guri A, Griffiths M, Khursigara CM, Corredig M (2012) The effect of milk fat globules on adherence and internalization of Salmonella Enteritidis to HT-29 cells. J Dairy Sci 95: 6937–6945. 23021758 10.3168/jds.2012-5734

[18] Inagaki M, Nagai S, Yabe T, Nagaoka S, Minamoto N, et al (2010) The bovine lactophorin C-terminal fragment and PAS6/7 were both potent in the inhibition of human rotavirus replication in cultured epithelial cells and the prevention of experimental gastroenteritis. Biosci Biotechnol Biochem 74: 1386–1390. 20622446 10.1271/bbb.100060

[19] Moore JB, Weeks ME (2011) Proteomics and systems biology: current and future applications in the nutritional sciences. Adv Nutr 2: 355–364. 22332076 10.3945/an.111.000554 PMC3125684

[20] Bianchi L, Puglia M, Landi C, Matteoni S, Perini D, et al (2009) Solubilization methods and reference 2-DE map of cow milk fat globules. J Proteomics 72: 853–864. 19111954 10.1016/j.jprot.2008.11.020

[21] Picariello G, Ferranti P, Mamone G, Klouckova I, Mechref Y, et al (2012) Gel-free shotgun proteomic analysis of human milk. J Chromatogr A 1227: 219–233. 22277183 10.1016/j.chroma.2012.01.014

[22] Spertino S, Cipriani V, De Angelis C, Giuffrida MG, Marsano F, et al (2012) Proteome profile and biological activity of caprine, bovine and human milk fat globules. Mol Biosyst 8: 967–974. 22193558 10.1039/c2mb05400k

[23] Liao Y, Alvarado R, Phinney B, Lonnerdal B (2011) Proteomic characterization of human milk fat globule membrane proteins during a 12 month lactation period. J Proteome Res 10: 3530–3541. 21714549 10.1021/pr200149t

[24] Yang B, Wang J, Tang B, Liu Y, Guo C, et al (2011) Characterization of bioactive recombinant human lysozyme expressed in milk of cloned transgenic cattle. PLoS One 6: e17593. 21436886 10.1371/journal.pone.0017593 PMC3059212

[25] Yang P, Wang J, Gong G, Sun X, Zhang R, et al (2008) Cattle mammary bioreactor generated by a novel procedure of transgenic cloning for large-scale production of functional human lactoferrin. PLoS One 3: e3453. 18941633 10.1371/journal.pone.0003453 PMC2565487

[26] Wang J, Yang P, Tang B, Sun X, Zhang R, et al (2008) Expression and characterization of bioactive recombinant human alpha-lactalbumin in the milk of transgenic cloned cows. J Dairy Sci 91: 4466–4476. 19038921 10.3168/jds.2008-1189

[27] Zhang R, Guo C, Sui S, Yu T, Wang J, et al (2012) Comprehensive assessment of milk composition in transgenic cloned cattle. PLoS One 7: e49697. 23185411 10.1371/journal.pone.0049697 PMC3504162

[28] Wisniewski JR, Zougman A, Nagaraj N, Mann M (2009) Universal sample preparation method for proteome analysis. Nat Methods 6: 359–362. 19377485 10.1038/nmeth.1322

[29] Nesvizhskii AI, Keller A, Kolker E, Aebersold R (2003) A statistical model for identifying proteins by tandem mass spectrometry. Anal Chem 75: 4646–4658. 14632076 10.1021/ac0341261

[30] Keller A, Nesvizhskii AI, Kolker E, Aebersold R (2002) Empirical statistical model to estimate the accuracy of peptide identifications made by MS/MS and database search. Anal Chem 74: 5383–5392. 12403597 10.1021/ac025747h

[31] Huang da W, Sherman BT, Lempicki RA (2009) Bioinformatics enrichment tools: paths toward the comprehensive functional analysis of large gene lists. Nucleic Acids Res 37: 1–13. 19033363 10.1093/nar/gkn923 PMC2615629

[32] D’Amato A, Bachi A, Fasoli E, Boschetti E, Peltre G, et al (2009) In-depth exploration of cow’s whey proteome via combinatorial peptide ligand libraries. J Proteome Res 8: 3925–3936. 19499900 10.1021/pr900221x

[33] Aziz A, Zhang W, Li J, Loukas A, McManus DP, et al (2011) Proteomic characterisation of Echinococcus granulosus hydatid cyst fluid from sheep, cattle and humans. J Proteomics 74: 1560–1572. 21362505 10.1016/j.jprot.2011.02.021

[34] Affolter M, Grass L, Vanrobaeys F, Casado B, Kussmann M (2010) Qualitative and quantitative profiling of the bovine milk fat globule membrane proteome. J Proteomics 73: 1079–1088. 19944786 10.1016/j.jprot.2009.11.008

[35] Lu J, Boeren S, de Vries SC, van Valenberg HJ, Vervoort J, et al (2011) Filter-aided sample preparation with dimethyl labeling to identify and quantify milk fat globule membrane proteins. J Proteomics 75: 34–43. 21907314 10.1016/j.jprot.2011.07.031

[36] Addis MF, Pisanu S, Ghisaura S, Pagnozzi D, Marogna G, et al (2011) Proteomics and pathway analyses of the milk fat globule in sheep naturally infected by Mycoplasma agalactiae provide indications of the in vivo response of the mammary epithelium to bacterial infection. Infect Immun 79: 3833–3845. 21690237 10.1128/IAI.00040-11 PMC3165467

[37] Mowbray AL, Kang DH, Rhee SG, Kang SW, Jo H (2008) Laminar shear stress up-regulates peroxiredoxins (PRX) in endothelial cells: PRX 1 as a mechanosensitive antioxidant. J Biol Chem 283: 1622–1627. 18024958 10.1074/jbc.M707985200

[38] Lee KA, Hammerle LP, Andrews PS, Stokes MP, Mustelin T, et al (2011) Ubiquitin ligase substrate identification through quantitative proteomics at both the protein and peptide levels. J Biol Chem 286: 41530–41538. 21987572 10.1074/jbc.M111.248856 PMC3308864

[39] Takahashi Y, Itoh F, Oohashi T, Miyamoto T (2003) Distribution of apolipoprotein E among lipoprotein fractions in the lactating cow. Comp Biochem Physiol B Biochem Mol Biol 136: 905–912. 14662312 10.1016/j.cbpc.2003.09.004

[40] Parker P, Sando L, Pearson R, Kongsuwan K, Tellam RL, et al (2010) Bovine Muc1 inhibits binding of enteric bacteria to Caco-2 cells. Glycoconj J 27: 89–97. 19936918 10.1007/s10719-009-9269-2

[41] Mahley RW, Innerarity TL, Rall SC Jr, Weisgraber KH (1984) Plasma lipoproteins: apolipoprotein structure and function. J Lipid Res 25: 1277–1294. 6099394

[42] Viturro E, Farke C, Meyer HH, Albrecht C (2006) Identification, sequence analysis and mRNA tissue distribution of the bovine sterol transporters ABCG5 and ABCG8. J Dairy Sci 89: 553–561. 16428624 10.3168/jds.S0022-0302(06)72118-X

[43] Bionaz M, Loor JJ (2008) ACSL1, AGPAT6, FABP3, LPIN1, and SLC27A6 are the most abundant isoforms in bovine mammary tissue and their expression is affected by stage of lactation. J Nutr 138: 1019–1024. 18492828 10.1093/jn/138.6.1019

[44] Yonezawa T, Haga S, Kobayashi Y, Katoh K, Obara Y (2009) Saturated fatty acids stimulate and insulin suppresses CIDE-A expression in bovine mammary epithelial cells. Biochem Biophys Res Commun 384: 535–539. 19427838 10.1016/j.bbrc.2009.05.012

[45] Spitsberg VL, Matitashvili E, Gorewit RC (1995) Association and coexpression of fatty-acid-binding protein and glycoprotein CD36 in the bovine mammary gland. Eur J Biochem 230: 872–878. 7541353 10.1111/j.1432-1033.1995.tb20630.x

[46] Sharp JA, Lefevre C, Nicholas KR (2008) Lack of functional alpha-lactalbumin prevents involution in Cape fur seals and identifies the protein as an apoptotic milk factor in mammary gland involution. BMC Biol 6: 48. 18986549 10.1186/1741-7007-6-48 PMC2600633

